# An *ex vivo* functional biomarker of treatment response in pediatric low-grade glioma

**DOI:** 10.1371/journal.pone.0331423

**Published:** 2026-03-05

**Authors:** Nichole M. Artz, Breanna Mann, Aaron Ebbs, Rami Darawsheh, Rajaneekar Dasari, Adebimpe Adefolaju, Noah Bell, Dimitri Trembath, Dominique Higgins, Scott Elton, Albert Baldwin, Shawn Hingtgen, David E. Kram, Andrew B. Satterlee

**Affiliations:** 1 Division of Pediatric Hematology-Oncology, Department of Pediatrics, University of North Carolina at Chapel Hill, Chapel Hill, North Carolina, United States of America; 2 Division of Pharmacoengineering and Molecular Pharmaceutics, Eshelman School of Pharmacy, University of North Carolina at Chapel Hill, Chapel Hill, North Carolina, United States of America; 3 Eshelman Innovation, University of North Carolina at Chapel Hill, Chapel Hill, North Carolina, United States of America; 4 Lineberger Comprehensive Cancer Center, University of North Carolina at Chapel Hill, Chapel Hill, North Carolina, United States of America; 5 Department of Pathology and Laboratory Medicine, University of North Carolina at Chapel Hill, Chapel Hill, North Carolina, United States of America; 6 Department of Neurosurgery, University of North Carolina at Chapel Hill, Chapel Hill, North Carolina, United States of America; Roswell Park Cancer Institute, UNITED STATES OF AMERICA

## Abstract

Children with subtotally resected pediatric low-grade glioma (pLGG) often face multiple lines of treatment, which are seldom capable of eliminating the entire tumor. Genomics-based biomarkers are often used to select targeted therapies, but this paradigm only yields overall response rates of ~50%. Functional precision medicine (FPM), where patient-specific therapeutic efficacy is evaluated by directly treating individuals’ tumor outside their body, can predict individualized drug responses for some cancers, but pLGG is notoriously difficult to maintain outside the body, limiting development of FPM for pLGG. We describe the first platform that can maintain, treat, and analyze zero-passage pLGG tumor tissue *ex vivo*, facilitating FPM testing. Here, we tested pLGG tumors on our previously validated Screening Live Cancer Explants (SLiCE) platform, which allows engraftment and testing of diverse CNS tumor types atop organotypic brain slice cultures (OBSCs). After ensuring reproducible engraftment and maintenance of all three living pLGG tumor tissues on SLiCE, we measured MAPK pathway response to targeted therapies via immunoblotting. In the tested BRAF KIAA1549 fusion+ tumor, Western blot demonstrated maintenance of expected paradoxical MAPK upregulation in response to dabrafenib treatment. We then measured tumor response to targeted therapies on SLiCE. As expected, none of the BRAF KIAA1549 fusion+ pLGG tumors were sensitive to dabrafenib treatment. Two out of the three tumors demonstrated predicted sensitivity to trametinib, whereas one tumor did not. While no clinical correlates were measured in this proof-of-concept study, this mixed response to MEK inhibition on SLiCE is representative of heterogeneous real-world clinical responses. Together, these data demonstrate the feasibility of SLiCE to become a new functional biomarker of response in a tumor type where functional models are exceptionally rare, establishing a foundation for future individualized treatment strategies.

## Introduction

Pediatric low-grade gliomas (pLGG) are the most prevalent central nervous system (CNS) tumors in children [1]. They are a heterogeneous group of WHO grade 1 and grade 2 tumors arising from neural cells, including glial, neuronal, and mixed glioneuronal cells. While they carry excellent overall survival outcomes [2], they have poor progression-free survival rates, particularly in cases of subtotal resection [3]. Patients with incompletely resected pLGG often undergo multiple surgeries and receive several types of therapy over many years, with most therapies failing to destroy the entire tumor. These treatments often lead to debilitating long-term morbidities [4–6] and severely diminish quality-of-life. To more effectively treat pLGG, limit invasive procedures, and minimize number of treatments given, the field must develop a better method to predict the most effective treatment for each individual’s tumor.

Deficiencies in current biomarkers of response necessitate a change in the way pLGG treatment plans are developed. Currently, many pLGGs are being treated with inhibitors of the mitogen-activated protein kinase (MAPK) pathway, which is most often upregulated in these cancers via genetic alterations such as BRAF-V600E mutation and BRAF-KIAA 1549 fusion. Presence of these mutations suggests that kinase inhibitors like dabrafenib (BRAF inhibition) or trametinib (MEK inhibition) may be effective, but only ~50% of children with BRAF alterations benefit from these targeted therapies. These limitations in genomic tumor biomarkers as predictors of personalized treatment sensitivities are now fueling a shift toward functional precision medicine (FPM). FPM evaluates therapeutic efficacy by directly treating individuals’ living tumor tissue outside their body (after tumor biopsy or resection) to gauge patient-specific activity and response. Some FPM models are beginning to deliver encouraging data to guide matched patient outcomes [7–12], but pLGG tumor tissues are notoriously difficult to maintain outside the body [13, 14] limiting options to directly screen pLGG tumors for drug sensitivities *ex vivo*. The development of new preclinical models of pediatric low‑grade glioma (pLGG) remains profoundly constrained by several factors. First, pLGGs are exceptionally scarce and are often not biopsied or surgically resected; in cases where tissue is obtained, it is typically fixed in formalin by pathology, preventing its use in functional or culture‑based studies. Second, these tumors are intrinsically slow‑growing and minimally proliferative, making it extremely difficult to establish and maintain cell lines or xenograft models. Third, even when viable models are generated, they frequently fail to faithfully preserve the molecular, cellular, and phenotypic features of the original tumor, limiting their utility for mechanistic or translational research.

In this study, we describe the first evidence that our Screening Live Cancer Explants (SLiCE) platform can maintain, treat, and analyze personalized drug sensitivities of passage-zero pLGG tumor tissues *ex vivo*. Here, we engraft three passage-zero pLGG tumor tissues onto our SLiCE platform, which has been developed over the past decade as a robust functional drug screening platform that can test both fresh and cryopreserved patient tumor tissues [15–20]. We first ensured reproducible engraftment and maintenance of three living pLGG tumor tissues on SLiCE. We then measured maintenance of MAPK pathway responses to dabrafenib and trametinib by assaying the phosphorylation statuses of MEK and ERK via immunoblotting. Finally, we measured sensitivity of each pLGG tumor tissue to dabrafenib and trametinib on SLiCE using our four-day assay, correlating *ex vivo* patient tumor responses to dabrafenib and trametinib to the expected response based on molecular characterization alone. The three pLGG tumors studied here represent the only pLGG specimens our team has collected across several years of tumor accrual from consenting patients at UNC Hospitals, underscoring the rarity of securing living pLGG tumors for research; our ability to maintain and test all three tumors on SLiCE overcomes a significant technical hurdle and positions SLiCE as a unique platform to one day guide treatment for children with pLGG.

## Materials and methods

### Experimental model and subject details

#### Animal ethics statement.

This study was carried out in strict accordance with the recommendations in the Guide for the Care and Use of Laboratory Animals of the National Institutes of Health. All animal work was approved by the Institutional Animal Care and Use Committee at the University of North Carolina-Chapel Hill under protocol 22–171. Rat pups were necessarily and rapidly euthanized by decapitation using a small-animal guillotine without prior anesthesia, as the use of anesthetics such as isoflurane significantly compromises neuronal health and function in brain tissue required for slice culture. This method was selected to meet scientific objectives and is consistent with AVMA Guidelines for the Euthanasia of Animals. The procedure was performed swiftly to minimize distress and alleviate suffering, with pups manually restrained or placed in a plastic cone to prevent movement and ensure a clean, humane decapitation. P8 Sprague-Dawley rat pups (RRID:MGI:5651135) were used exclusively for OBSC preparation based upon previous optimization [15]. Animals were housed with one mother and no more than ten pups. All animals were euthanized prior to weaning. Adult dams were euthanized in a CO₂ chamber following completion of pup collection, and bilateral thoracotomy was performed after apparent death to confirm euthanasia. Efforts to alleviate suffering included continuous observation of all animals prior to euthanasia. Any pup or adult exhibiting signs of distress, such as loss of feeding or drinking behavior, excessive quietness, lack of grooming, seizures, or a body condition score (BCS) of 2 or less, was immediately euthanized using CO₂ followed by cervical dislocation. All procedures were carried out promptly to minimize pain and stress.

#### Human subjects ethics statement.

All brain tumor specimens were collected at University of North Carolina Hospitals. Informed consent was obtained under IRB-approved protocols 20–1878 or 23–0834. CNS tumor patients were screened during the accrual window. Three pLGG specimens with a confirmed WHO diagnosis as a pLGG were consented and produced viable tissue aliquots met pre-engraftment requirements (quantity ≥50 mg). All samples were coded through an honest broker. Laboratory results were not returned to clinicians, and research personnel were blinded to clinical outcomes until experiments and data reduction were complete. A total of three patients were included in the present study, two females and one male, with ages ranging between 4 and 8 years old.

### Method details

#### Chemicals.

Dabrafenib (AMBH47A8ED73), reconstituted in DMSO at 192.47 mM, and trametinib (AMBH2D6F887E), reconstituted in DMSO at 35.75 mM, were purchased from Sigma-Aldrich.

#### Lentiviral vectors.

mCherry protein fused to firefly luciferase (LV–mCh-FL) were used in this study.

#### Preparing patient tissue for liquid nitrogen storage.

Tissue was prepared using the protocol previously described [15]. Immediately after brain tumor tissue was surgically resected, tissue was placed in sterile 4°C Neurobasal-A medium. The resected tumor tissue was manually minced into approximately 0.5 mm pieces by scalpel and washed with PBS. Tissue was then placed in a cryogenic vial in CryoStor CS10 at a concentration of 150 mg/mL and frozen at −80°C for 24 hours in a Mr. Frosty prior to being transferred into liquid nitrogen.

#### Generating OBSCs.

OBSCs were generated using the protocol previously described [15]. Briefly, P8 Sprague-Dawley rat pups were dissected and the brain was isolated. 300 um thick coronal OBSCs were sliced using a vibratome and placed onto Millicell culture inserts with 1mL brain slice media (BSM) comprised of Neurobasal-A medium supplemented with heat-inactivated pig serum, heat-inactivated rat serum, L-glutamine, KCl, HEPES, sodium pyruvate and penicillin-streptomycin, added under each well. Two separate OBSCs are placed on each transwell insert in a 6-well plate. Plates were then transferred to 37°C incubator with 5% CO2, and 95% humidity. Representative OBSCs from each batch are screened for viability on Day 4 after generation using our previously established propidium iodide fluorescence assay [15]. All OBSCs used in this manuscript passed our quality control metric (> 80% OBSC viability).

#### Preparing tissue for engraftment onto OBSCs.

Preparing tissue for engraftment onto OBSCs was carried out using the protocol previously described [15]. Briefly, the preserved brain tumor tissue was thawed, then filtered through 100 mm cell strainer and incubated with Lentivirus and polybrene for two hours, washed with PBS to remove excess virus, and reconstituted in Neurobasal-A. Following the two-hour incubation period in Lentivirus and polybrene, the tissue was then engrafted onto OBSCs at 0.5 mg tissue in 2mL Neurobasal-A with a microtumor on each hemisphere of the OBSC. The 6-well plate was subsequently incubated at 37°C for the remainder of the assay. Viability of tissue is assessed as day 4 bioluminescence signal reaching the predetermined minimum threshold [21].

#### Dosing screening with OBSCs.

Dosing was completed following the standard protocol previously described [15]. Briefly, 24 hours after tissue engraftment onto OBSCs wells with microtumors were randomized to minimize seeding effects, the media beneath each transwell was replaced with 1 mL of drug diluted with BSM, resulting in an n of 4 per dose. Dose ranges were based on past *in vitro* results [22, 23] and OBSC toxicity, and doses were as follows: trametinib ranged from 0 to 100µM, and dabrafenib ranged from 0 to 10µM. 96 hours after tumor seeding, the tumor bioluminescence was measured utilizing an AMI optical imager.

#### Western blot analysis.

Tumors were carefully excised from tumor-bearing OBSCs and snap frozen at 80°C on days 2 and 4 after tumor engraftment. Samples were thawed and suspended in complete lysis buffer (RIPA buffer), then incubated on ice for 5 minutes. Samples were then sonicated in water bath for 5 cycles of 30 seconds on/30 seconds off, then centrifuged at 13,000 rpm for 10 minutes at 4°C. Protein concentration was determined in each sample using Bradford assay BioRad Reagent and read on VersaMax plated reader. Protein concentration was then adjusted to 1–2 µg/µL in 1x Laemmli Sample Buffer. Samples were heated and run on gel at 120V for 60–90 minutes, then transferred to Nitrocellulose membrane using BioRad turbo blot transfer system. Ponceau solution was used to visualize protein, blots were blocked and agitated for 1 hour at room temperature, then incubated overnight in primary antibody (1:1000) solution at 4°C. Blots were washed a total of 5 times, incubated with secondary antibodies, washed again, and finally, BioRad Clarity ECL solution was used to visualize protein bands on Bio Rad Chemidoc MP imaging system. Uncropped, minimally adjusted original blots underlying Fig 2 are provided as Supporting Information (S1 Fig Raw Blot and Gel Images).

#### Statistical analysis.

Mean values between two groups were compared using Welch’s two-tailed t tests. The p values are represented as follows: ****p < 0.0001, ***p < 0.001, **p < 0.01, *p ≤ 0.05, and not statistically significant when p > 0.05. The Robust Regression and Outlier Removal (ROUT) method was used to identify outliers from the day 4 microtumor replicates. A *Q-*value of 1% was used to determine significance. All statistical analyses were performed using GraphPad Prism (version 10.4.1).

## Results

### Tumor characterization

In this study, tumor tissue was obtained from three pLGG tumors resected between March 2023 and January 2024. The cohort included two female patients and one male patient. Two of the three tumors were gross-totally resected during initial surgery, and none of the patients received tumor-directed therapy following resection. Clinical immunohistochemical analysis confirmed the diagnosis of pLGG, specifically pilocytic astrocytoma, WHO grade 1, for all tumors. BRAF-KIAA 1549 fusion was detected in each tumor for clinical purposes via next generation sequencing customized FusionPlex Solid Tumor kid for Illumina and FusionPlex Supplement for Illumina. None of the tumors harbored the oncogenic BRAF-V600E mutation. As of March 2025, at initial manuscript submission, there have been no documented recurrences/progressions. Tumor and patient demographics are listed in Table 1.

**Table 1:** Demographics of pediatric low-grade glioma (pLGG) patients.

Demographics	pLGG-1	pLGG-2	pLGG-3
**Age at diagnosis**	6 years	8 years	4 years
**Sex**	Male	Female	Female
**Tumor location**	Posterior fossa	Posterior fossa	Posterior fossa
**Initial resection**	Subtotal resection	Gross total resection	Gross total resection
**Histological diagnosis**	Pilocytic astrocytoma CNS WHO grade 1	Pilocytic astrocytoma CNS WHO grade 1	Pilocytic astrocytoma CNS WHO grade 1
**Genomic alteration**	BRAF:KIAA 1549^+^	BRAF:KIAA 1549^+^	BRAF:KIAA 1549^+^
**Treatment**	Surgical resection plus observation	Surgical resection plus observation	Surgical resection plus observation
**Recurrence**	None to date	None to date	None to date

### Tissue seeding and survival on OBSCs

**Fig 1:**
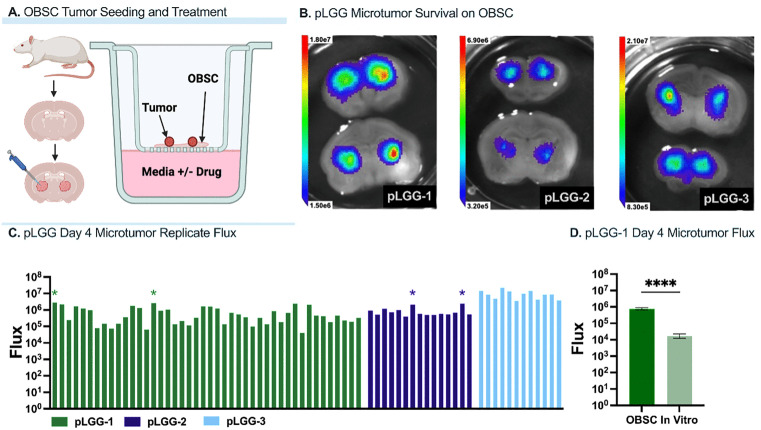
(a) Workflow for organotypic brain slice culture (OBSC) slicing, tumor tissue seeding, and drug treatment. (b) Live tumor cell bioluminescence four days post tumor tissue seeding on OBSCs. (c) pLGG microtumor replicate viability measured via bioluminescent flux, with four statistical outliers showing increased viability compared to the average survival of each microtumor (*statistical outlier measured using ROUT method, Prism). (d) pLGG-1 viability on OBSC compared to *in vitro*, showing a 43-fold increase in survival on OBSC (****p < 0.0001 compared to pLGG-1 microtumors *in vitro* using Welch’s t test, Prism).

We first tested the reproducible engraftment and survival of all three pLGG tumor tissues on SLiCE, which uses living organotypic brain slice cultures (OBSCs) from P8 Sprague-Dawley rat pups as living tissue substrates on which to engraft and treat patient tumor tissues. 0.5 mg of mCh-FLuc-transfected pLGG tumor tissue was seeded atop each hemisphere of each freshly prepared, coronally sectioned OBSC (Fig 1A). Live tumor cell bioluminescence imaging was performed four days post-seeding (the standard length of our SLiCE assay) and confirmed reproducible pLGG tumor cell survival (Fig 1B). pLGG microtumors were reproducibly established from every seeded replicate, with only four statistical outliers showing *increased* viability compared to the average survival of each microtumor. In contrast, pLGG tissue seeded in standard *in vitro* culture showed 43-fold less survival four days after engraftment (p < 0.0001 compared to pLGG microtumors on OBSCs, Fig 1C). To our knowledge, this is the first evidence that passage-zero pLGG tumor tissue can be maintained *ex vivo.*

### Western blot analysis of treated pLGG on OBSC

**Fig 2:**
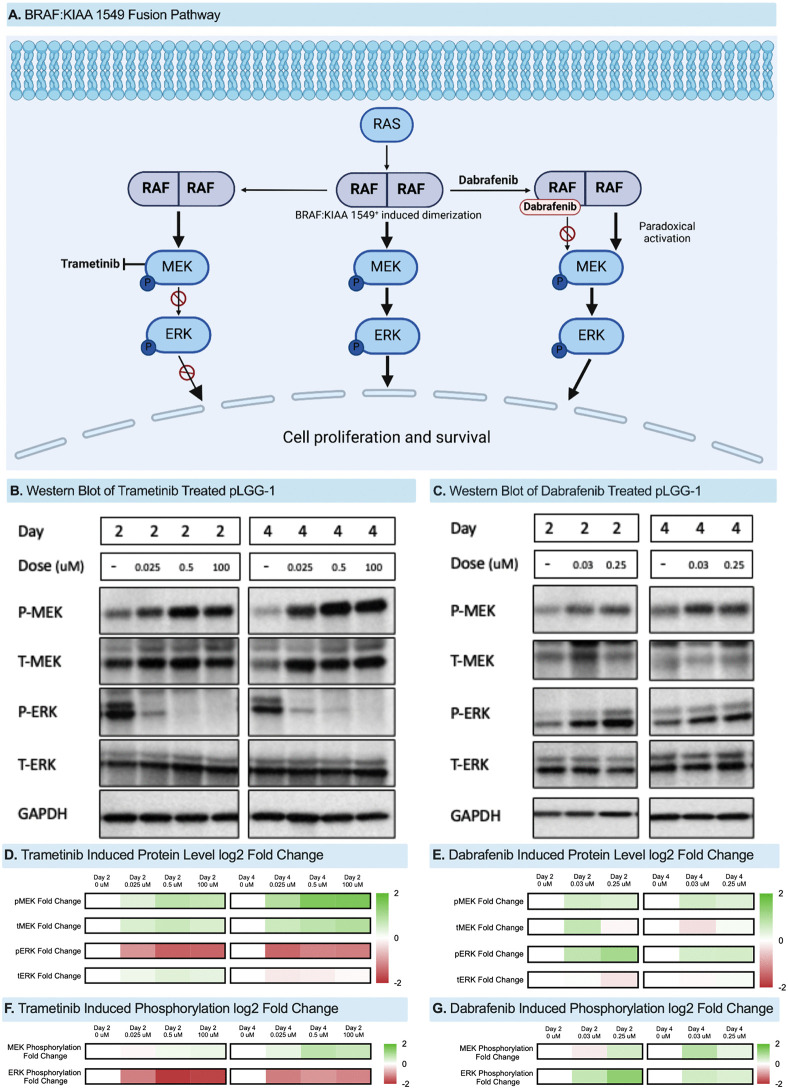
(a) RAS-MAPK pathway demonstrating the effects of the BRAF:KIAA 1549 fusion and targeted therapy with dabrafenib and trametinib. (b) Western blot of BRAF:KIAA 1549 + pLGG-1 cells seeded on organotypic brain slice cultures and treated with trametinib. (c) Western blot of BRAF:KIAA 1549 + pLGG-1 cells seeded on OBSCs and treated with dabrafenib. (d) Heatmap of trametinib treatment-induced protein levels of pMEK, tMEK, pERK, and tERK after 24 and 72 hours. (e) Heatmap of dabrafenib treatment-induced protein levels of pMEK, tMEK, pERK, and tERK after 24 and 72 hours. (f) Heatmap of normalized band intensities (pERK/tERK and pMEK/tMEK) for trametinib treatment at 24 h and 72 h. (g) Heatmap of normalized band intensities (pERK/tERK and pMEK/tMEK) for dabrafenib treatment at 24 h and 72 h.

To evaluate whether OBSC-engrafted pLGG tissue maintained expected MAPK signaling behavior, we performed western blot analysis following targeted therapy. The BRAF:KIAA1549 fusion is known to produce distinct responses to MEK and BRAF inhibition: the MEK inhibitor trametinib typically suppresses MAPK signaling and reduces cell survival, whereas the BRAF inhibitor dabrafenib paradoxically activates MAPK signaling through RAF dimerization and altered phosphorylation dynamics (Fig 2A) [24–27]. OBSC-engrafted pLGG-1 was treated with trametinib or dabrafenib for 24 or 72 hours, and phosphorylation of MEK and ERK was quantified by immunoblotting (Fig 2B-C).

To quantify treatment-induced changes, phosphorylated and total MEK and ERK levels were measured and normalized (Fig. 2D–E). Trametinib treatment resulted in increased pMEK and tMEK, accompanied by a reduction in pERK, while tERK remained stable (Fig. 2D). In contrast, dabrafenib did not induce decreases in pMEK or pERK, even paradoxically increasing pERK by up to 0.9-log2 fold (Fig. 2E). We then calculated phosphorylation ratios for pMEK/tMEK and pERK/tERK, enabling direct comparison of pathway modulation across treatments. Trametinib treatment reduced pERK/tERK phosphorylation by up to 1.6-log2 fold, whereas dabrafenib induced a modest paradoxical increase of approximately 1.2-log2 fold (tERK was also slightly elevated after treatment). pMEK/tMEK levels rose modestly following both treatments (up to 0.7-log2 fold with trametinib and 0.7-log2 fold with dabrafenib), reflecting pathway modulation at or upstream of MEK. These findings demonstrate that BRAF:KIAA1549 fusion-positive pLGG-1 retains its expected molecular response to therapy on the OBSC platform, enabling direct measurement of clinically relevant signaling responses in a living, patient-derived context.

### Tumor sensitivity to targeted therapeutics

**Fig 3:**
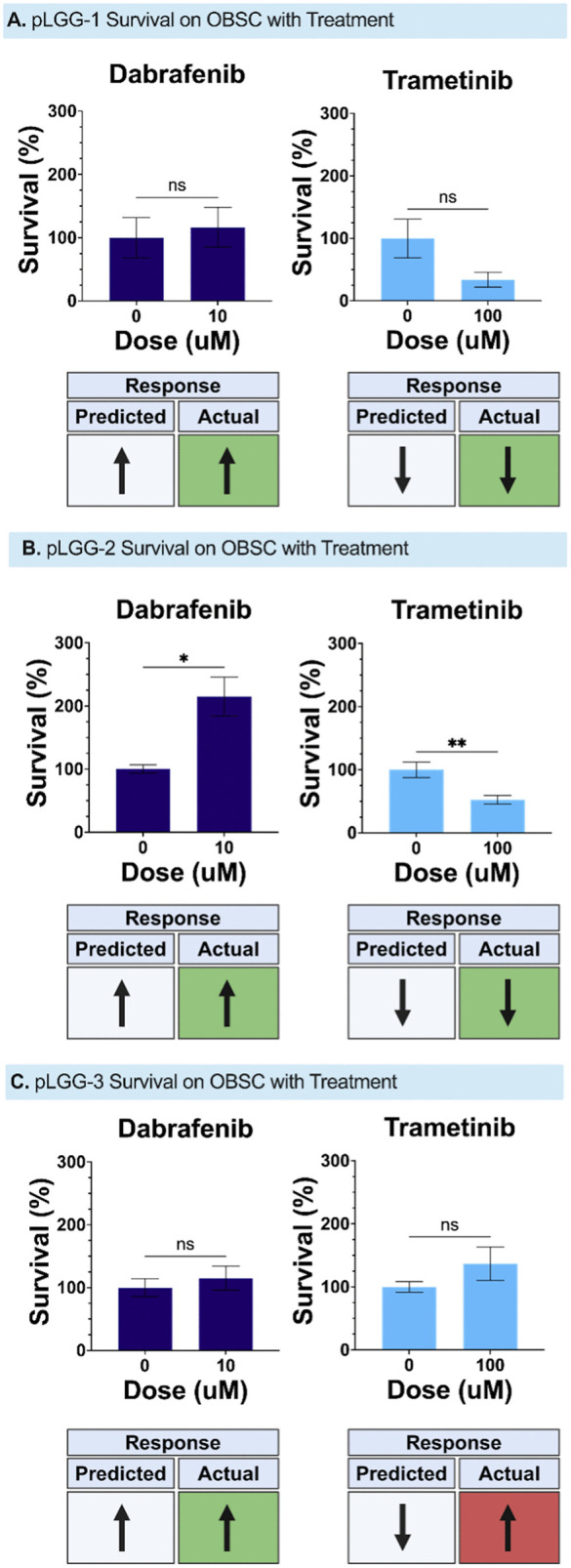
Tumor response to treatment with dabrafenib and trametinib. PLGG-1 (a) and pLGG 2 (b) demonstrated tumor cell growth in response to dabrafenib treatment and tumor cell death in response to trametinib treatment. PLGG-3 (c) demonstrated tumor cell growth in response to dabrafenib and trametinib treatment (*p < 0.05, **p < 0.01 using Welch’s t test, Prism).

Finally, we evaluated the responses of pLGG tumor tissues to treatment with dabrafenib and trametinib on OBSCs (Fig 3). Based on all tumors’ BRAF:KIAA1549^+^ status, we expected dabrafenib to be ineffective in all tumors and for trametinib to induce tumor suppression in all tumors – however, historical clinical response data suggests that mutational status does not always predict antitumor efficacy [28]. As predicted by the molecular signaling measured in Fig 2, dabrafenib was not effective against pLGG-1, but trametinib induced 68% tumor kill 3 days after treatment (Fig 3A). Similarly, dabrafenib induced tumor growth in pLGG-2 while trametinib induced significant tumor kill (Fig 3B). Interestingly, while dabrafenib was again ineffective against pLGG-3, trametinib was also ineffective, contradicting the molecular test and suggesting that other treatment resistance mechanisms may be present in this tumor which could make treatment in the clinical setting less effective (Fig 3C).

Fortunately for all three of these patients, no treatments were prescribed after surgery. This hindered our ability to correlate our laboratory results with post-treatment patient outcomes, but because progression free survival is as low as 45% with subtotal-resection [29], necessitating effective adjuvant treatment, we believe the data presented here represent a significant step toward validating a functional precision medicine platform for this rare tumor type with few representative *ex vivo* models in existence.

## Discussion

This timely work addresses a critical gap by enabling functional testing in a tumor type where viable models are extremely difficult to obtain and maintain. Static, molecular biomarkers such as BRAF-V600E and BRAF-KIAA 1549 fusion alterations are driving treatment guidance in pLGG, but these may not be the only drivers of tumor growth and proliferation within a single patient’s tumor. Tovorafenib, a second generation RAF inhibitor, and combination dabrafenib and trametinib have both received FDA approval in the past two years [28, 30–37], and as ongoing clinical trials continue to shed more light on efficacy and safety, especially compared to conventional chemotherapies [38–40], patients with seemingly identical tumors often experience opposite responses to these targeted agents. To supplement these static biomarkers and more accurately predict a patient’s response to treatment, we describe here a new *functional* paradigm to predict pLGG response: direct *ex vivo* killing of the tumor tissue itself.

Remarkably, each of these three patient-derived models demonstrated consistent engraftment and survival on SLiCE. The larger quantity of pLGG-1 tissue received enabled us to conduct a greater number of experiments, including confirming the *functional* persistence of the BRAF fusion event by demonstrating downstream MAPK inhibition by a MEK inhibitor and paradoxical downstream upregulation by a first-generation BRAF inhibitor. Finally, and most importantly, while all tumors expressed BRAF KIAA1549 fusion, which predicts response to MEK inhibition, only two out of three pLGG tumors responded to *ex vivo* trametinib treatment on SLiCE. This variable sensitivity aligns more closely with the clinical experience of heterogeneous response to MEK inhibition across all BRAF KIAA1549^+^ pLGG. These data underscore the importance of functional precision medicine platforms like SLiCE, particularly for rare tumors with limited preclinical models and for tumors like pLGG-3 which do not respond to treatment according to their purported driver mutational status.

pLGG treatment on SLiCE can also have significant implications in preclinical drug development. A major obstacle in translating novel drugs and drug combinations to treat pLGG remains the fidelity of the limited pLGG preclinical models in existence. Patient-derived pLGG cells are extremely difficult to serially passage in culture or in animals, which has led to the development of innovative but genetically inauthentic patient-derived *TP53-*null pLGG models [41–43], senescence-blocking models [44], and cell lines with uncommon co-occurring mutations such as CDKN2A [45]. While potentially useful for early-stage drug discovery [46], these models cannot capture pLGG patient-specific variability.

Furthermore, current preclinical models may overestimate the potency of some therapeutics compared to their clinical response rate. For example, in contrast to near-ubiquitous preclinical efficacy data of the second-generation RAF inhibitor, tovorafenib, against BRAF fusion-mutated cell lines and xenograft models [47], approximately 50% of children with relapsed pLGG receiving the drug do not experience an objective response by RAPNO criteria [28]. Together, this underscores a need for models like SLiCE to guide drug development for pLGG in the preclinical space as well as in the clinic [2, 13, 41, 48–51].

While the number of tumors tested is small, this reflects the real-world scarcity of pLGG specimens and highlights the feasibility of applying this approach under these constraints. None of the three patients have received any post-surgery chemotherapy, including trametinib or dabrafenib. The opportunity to correlate our *ex vivo* responses to individualized *in vivo* responses would further validate this novel pLGG model, and we continue to consent patients to our actively accruing clinical feasibility study (NCT05978557) to achieve this goal [52]. Additionally, while the presence of the BRAF fusion in the tumor specimen was confirmed prior to OBSC engraftment, we did not repeat this test following engraftment. We begin treatment just one day after tumor seeding and complete the assay just three days later, minimizing the chance for genetic drift in culture [15]. Lastly, this model lacks an intact blood-brain barrier and blood supply, which may limit fidelity in some contexts. However, the OBSC itself contains innate stromal and immune cells which react to the tumor [15], and the passage-zero tumor tissue contains both tumor and tumor-associated cells. We have observed that not only do tumors respond to anatomical features of the OBSCs on which they are engrafted, but the living cells within OBSCs also respond to the presence of tumor [15]. Fig 1 suggests the presence of the living microenvironment promotes survival just as we have seen in our previous studies [21]. Our current studies focus on how therapy impacts overall tumor survival, similar to the type of data an MRI could provide; future and ongoing research is focused on diving deeper into the different roles of tumor and tumor-associated cells on SLiCE and their individual responses to therapy.

Together, our findings highlight how SLiCE can become a new functional biomarker of pLGG response to treatment, uniquely capturing living, zero-passage, patient-specific pLGG tumor tissue, enabling drug sensitivity testing for therapeutics such as dabrafenib and trametinib in just four days, and yielding reproducible and patient-specific drug sensitivity results in a context where conventional models have largely failed.

## Supporting information

S1 Fig S1_raw_images. (PDF)

S2 Fig StrikingImage. (TIF)

S1 Date Supplemental Information – Raw Data. (XLSX)
