# Supplementary material for: An ex vivo functional biomarker of treatment response in pediatric low-grade glioma
Source: PLoS One. 2026 Mar 5;21(3):e0331423. doi: 10.1371/journal.pone.0331423 (PMC12962519; doi:10.1371/journal.pone.0331423)
Supplement: S1 Fig — (PDF) [file pone.0331423.s001.pdf]

A) Dabrafenib pERK

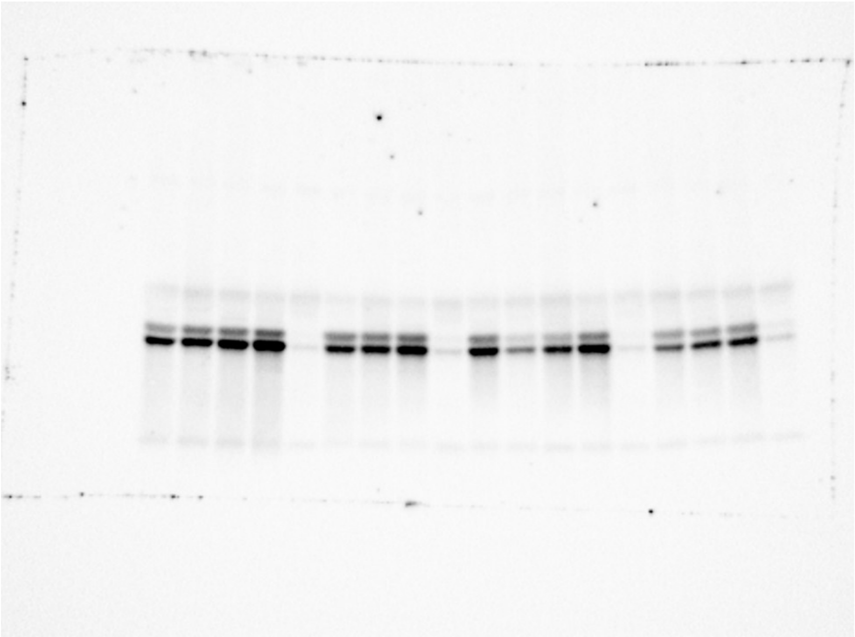

B) Dabrafenib tERK

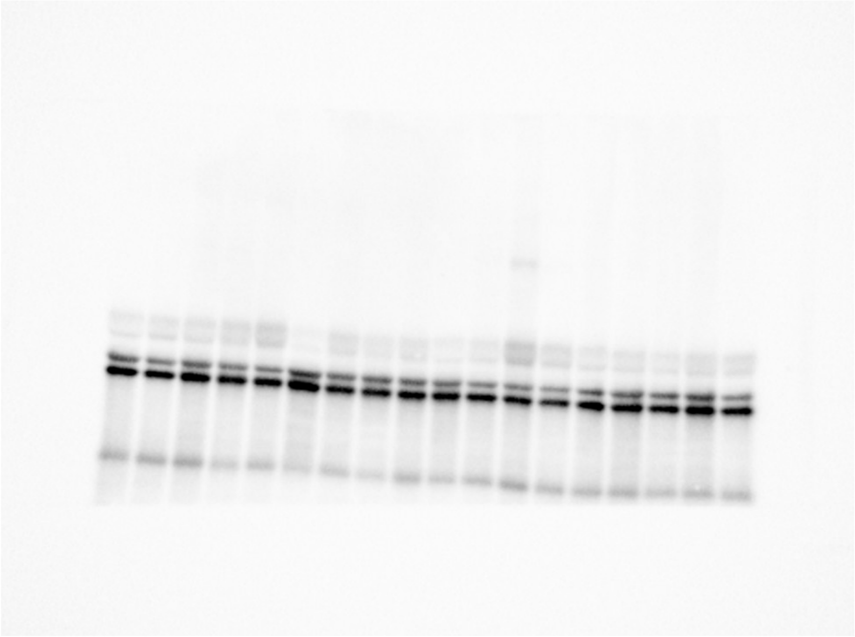

C) Dabrafenib pMEK

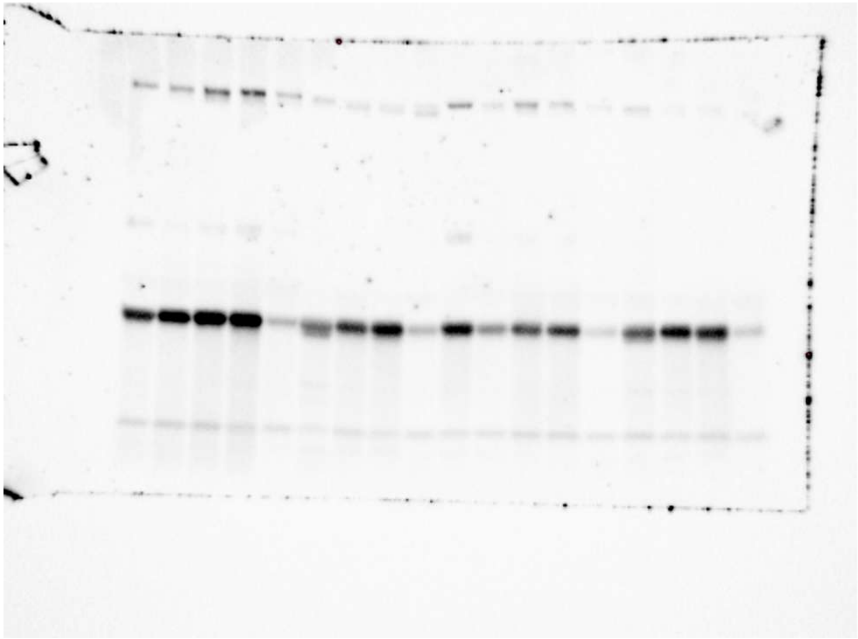

D) Dabrafenib tMEK

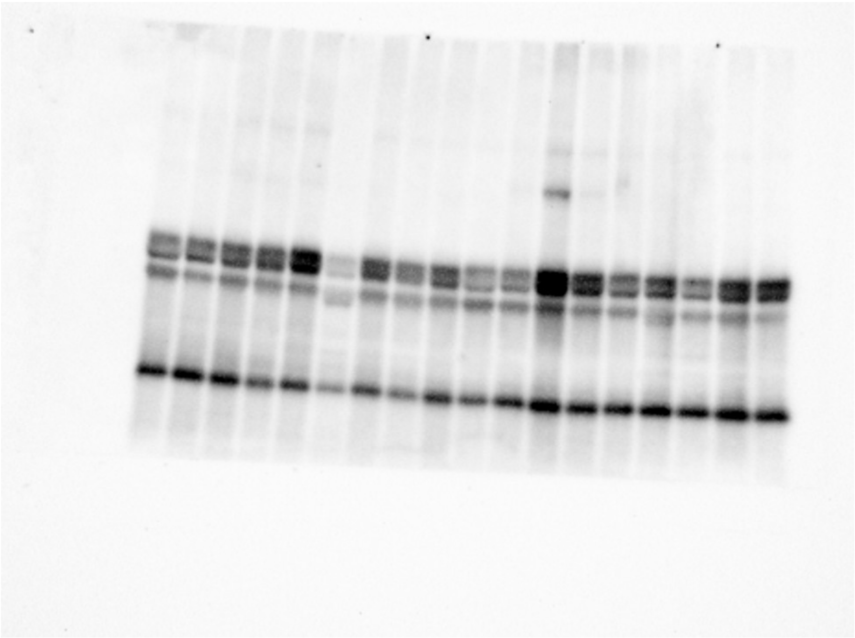

E) Dabrafenib GAPDH

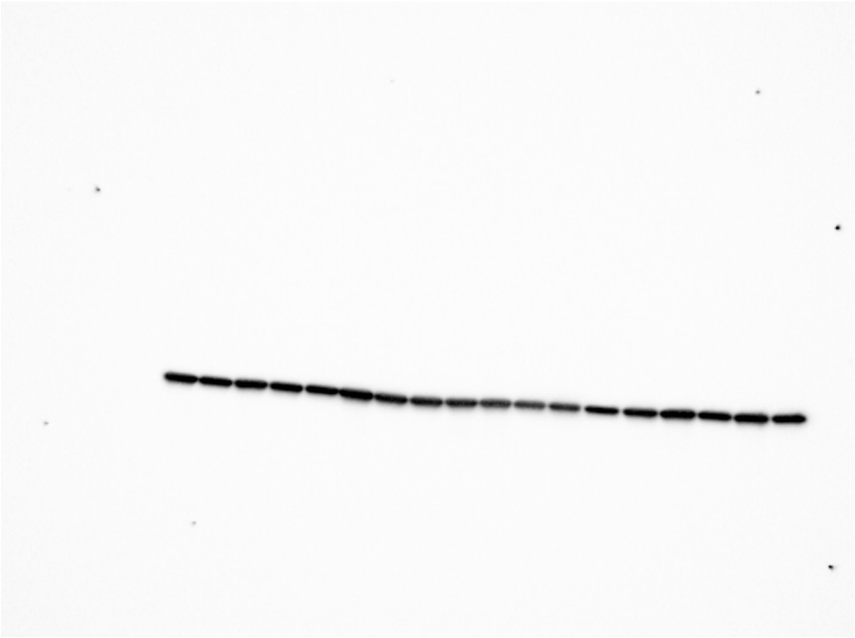

F) Trametinib pERK

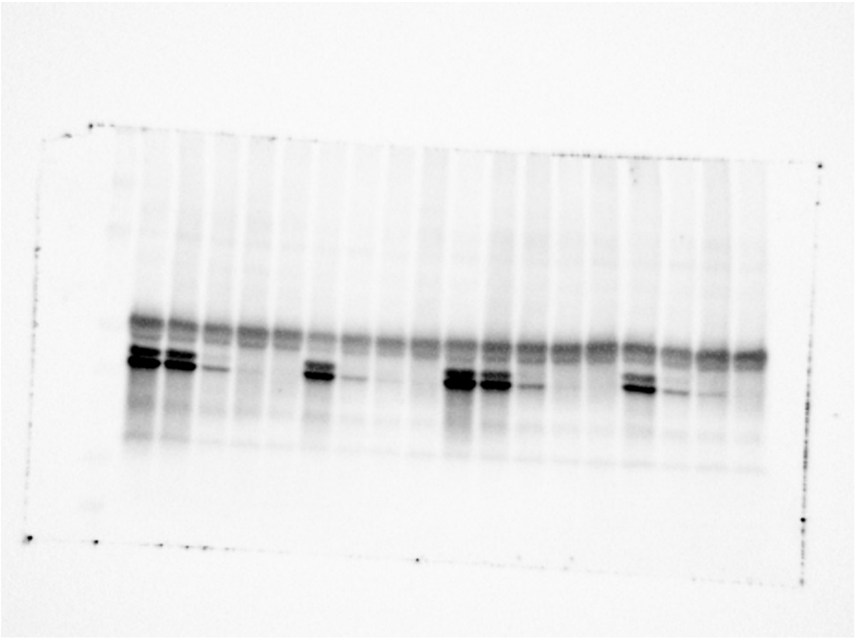

G) Trametinib tERK

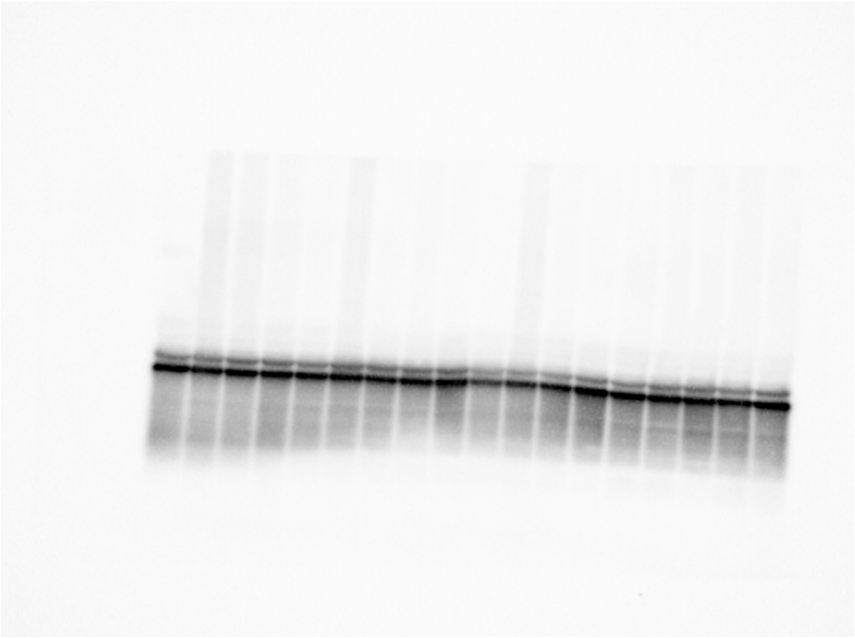

H) Trametinib pMEK

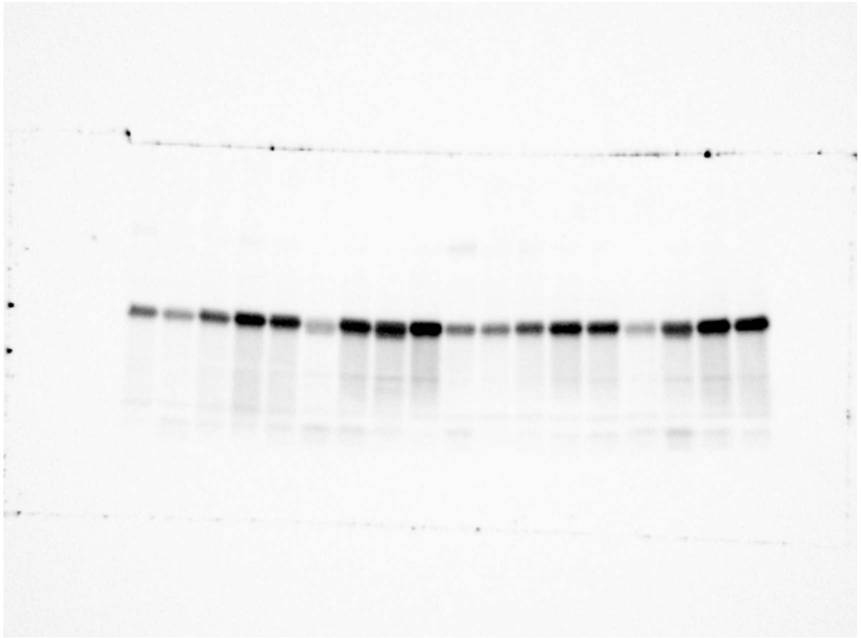

I) Trametinib tMEK

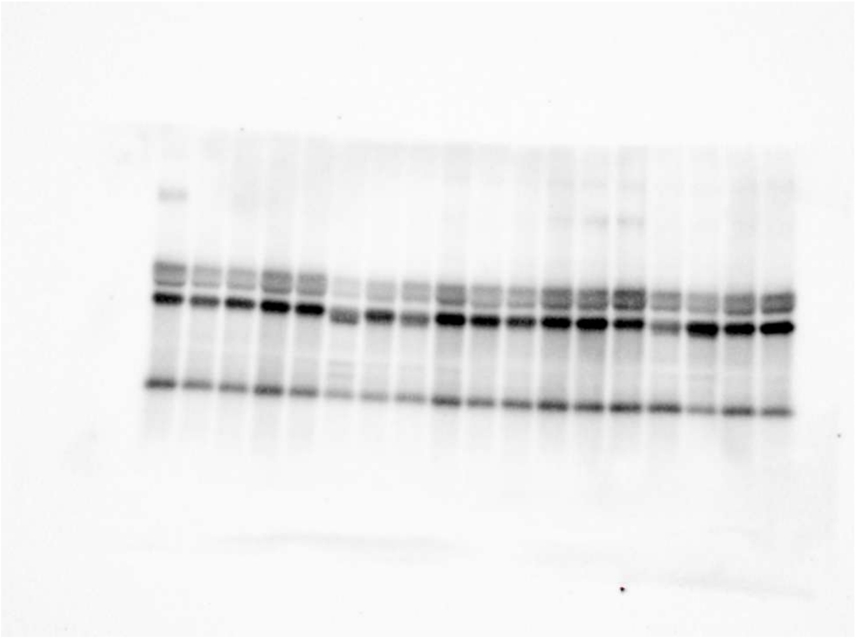

J) Trametinib GAPDH

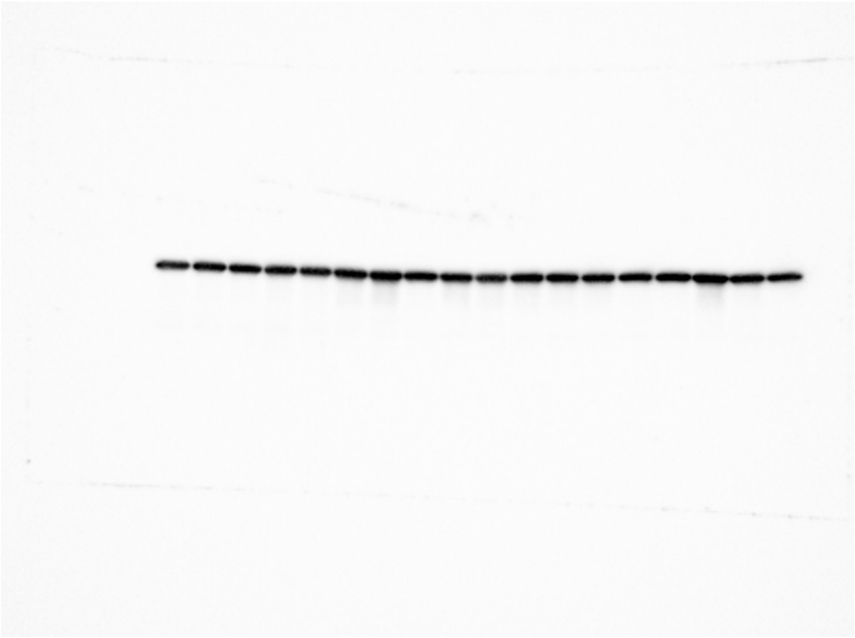

Figure S1: Raw unedited western blots. Data reported in this work is contained in Lanes 11-18 of each image.
